# Exploring the Ruminal Microbial Community Associated with Fat Deposition in Lambs

**DOI:** 10.3390/ani11123584

**Published:** 2021-12-17

**Authors:** Yukun Zhang, Xiaoxue Zhang, Fadi Li, Chong Li, Deyin Zhang, Xiaolong Li, Yuan Zhao, Weimin Wang

**Affiliations:** 1 College of Animal Science and Technology, Gansu Agricultural University, Lanzhou 730070, China; greenday.zyk@foxmail.com (Y.Z.); zhangxx@gsau.edu.cn (X.Z.); lichong@gsau.edu.cn (C.L.); lixl@st.gsau.edu.cn (X.L.); zhaoy@st.gsau.edu.cn (Y.Z.); 2 Engineering Laboratory of Sheep Breeding and Reproduction Biotechnology in Gansu Province, Minqin Zhongtian Sheep Industry Co., Ltd., Wuwei 733300, China; lifd@lzu.edu.cn; 3 The State Key Laboratory of Grassland Agro-Ecosystems, College of Pastoral Agriculture Science and Technology, Lanzhou University, Lanzhou 730020, China; zhangdy@st.gsau.edu.cn

**Keywords:** 16S rRNA, sheep, fat deposition, rumen, microbiota

## Abstract

**Simple Summary:**

In modern sheep production systems, less energy is required to gain lean tissue than to deposit fat; therefore, producers are attempting to decrease fat deposition costs by altering nutrient use to benefit the production of leaner carcasses. Microbes in the rumen have vital functions in feed digestion; however, limited research has been performed on the rumen microbiome’s effect on fat deposition. This study revealed variations in microbial populations in rumen carrying different fat deposition phenotypes in a characteristic way, and these findings could aid in developing strategies for manipulating rumen microbiota to alter the production performance of sheep.

**Abstract:**

Microbial communities of the sheep rumen have been studied extensively; however, their involvement in the regulation of fat deposition is unknown. Herein, we aimed to identify the correlations among fat deposition-related phenotypes and the effect of microbiota on changes in body fat accumulation. The rumen microbiota of 141 lambs was profiled by 16S ribosomal RNA sequencing, and the volatile fatty acids’ (VFAs’) concentrations were quantified by gas chromatography. Subsequently, the animals were grouped according to body mass index (BMI) to compare the microbiota of the rumen among the sheep with different fat deposition levels. Results further revealed differences in terms of the species abundance, diversity, and microbial composition between sheep with different fat deposition levels. Linear discriminant analysis (LDA) Effect Size (LEfSe) analysis and Random Forest (RF) regression analysis identified changes in 29 ruminal bacteria, which may be the main driver for different fat deposition.

## 1. Introduction

Fat-tailed sheep, representing 25% of the global sheep population [1], evolved from the wild ancestor of thin-tailed sheep approximately 5000 years ago. Domestication and long-term selection have led the fat-tailed sheep to show high adaptability to extreme environments, which, combined with a good fat deposition ability, represents an increase in the energy storage in the form of adipose tissue [2]. During periods of cold and food deprivation, body fat undergoes massive decomposition to provide energy for metabolism. This biological characteristic has been preserved through evolution and is not affected by nutritional quality, geographical environment, and other factors [3]. However, in modern sheep production systems, the use of intensive or semi-intensive feeding systems mean that fat is not an important energy source. Conversely, more energy is required for fat deposition than to produce the same amount of lean tissue and, thus, feed efficiency reduces accordingly [4]. Furthermore, people are becoming more aware of the unhealthy effects of a high amount of fat in meat and meat products, which causes obesity and associated metabolic pathologies [5]. Therefore, methods to modulate fat deposition in sheep are urgently required.

For ruminants, rumen microbial fermentation can produce energy-rich volatile fatty acids (VFAs) and microbial proteins, thereby facilitating nutrition absorption and energy harvesting [6]. The rumen microbiome composition also has been shown to have a substantial impact on growth performance and productivity in ruminants, and the changes in the rumen microbial population will ultimately form the animal’s phenotype and characteristics [7,8,9]. Notably, environmental factors, such as drug use and diet, have profound effects on the composition of the rumen microbiota. In view of this, modulation of rumen microbial composition offers an opportunity to improve host metabolism and regulate fat deposition. To date, research on this topic has mainly focused on model animals and other monogastric animals, with few studies conducted in ruminants, especially sheep. Thus, it is important to reveal the characteristics and functions of the sheep rumen microbiota associated with fat deposition.

Herein, the fat deposition-related phenotypes of 141 male lambs were measured and Spearman’s analysis was used to determine the correlations among the phenotypes to explore the basis of feature grouping. Next, we investigated the differences in rumen microbial community structure and potential function in lambs with different levels of fat deposition, using 16S ribosomal RNA (rRNA) gene sequencing and analysis. The findings of this study evaluated the correlation between microbiota and fat deposition, thereby providing insights into the development of effective approaches to manipulate fat deposition in ruminants.

## 2. Materials and Methods

### 2.1. Animals and Sampling

The present study used Male Hu lambs (*n* = 141, fat-tailed sheep). The lambs were housed in individual units (0.8 × 1.5 × 1.0 m) and had free access to corn–soybean meal-based diets (Table A1), which were prepared following the recommended feeding standards for sheep in China (NY/T816-2004). The lambs were fed twice daily (0900 and 1900 h) and had *ad libitum* access to water. The experiment comprised an adaptation period of 14 days and a data collection period of 100 days (during which the lambs were 80 to 180 days old). For all animals, their body weight (BW), body length, and feed consumption were recorded at the beginning (80 d) and the end of the experiment period (180 d), the results of which were used to calculate the average daily feed intake (ADFI), the body mass index (BMI), and the average daily gain (ADG).

**Figure A1:**
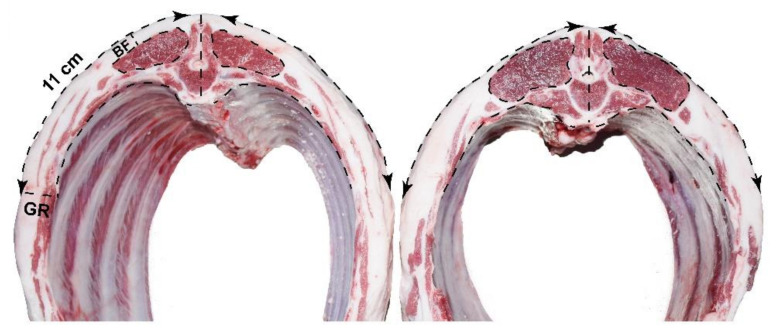
Rib thickness (GR value): The GR value is the thickness of the tissue between the 12th and 13th ribs, 11 cm from the mid-dorsal spine line, and is used to represent the fat content of the carcass (Figure 1). According to China Agricultural Industry Standard (NY/T 630-2002, Lamb and Mutton Evaluation and Grading), GR value is called rib thickness.

At the end of the experiment, all animals were slaughtered, and the animals’ live weights after fasting for 18 h were measured before slaughter. After slaughtering, the tail fat, perirenal fat, and mesenteric fat were extracted from their carcasses and measured (<5 min after slaughter). The relative fat weights (%) were calculated as 100 × fat weight/live weight, including the relative tail fat weight, the relative perirenal fat weight, and the relative mesenteric fat weight. During collection of total ruminal content, samples were immediately frozen in dry ice, transported to the laboratory in dry-ice packages, and stored at −20 °C before DNA analysis. The carcasses were stored at 4 °C for 24 h before manual segmentation. During cutting, the backfat thickness at the 12–13th ribs and GR value (rib thickness; Figure A1) were measured using digital vernier calipers. The concentration of VFAs in rumen chyme was analyzed using a TRACE-1300 series GC ultra-gas chromatograph (Thermo Scientific, Milan, Italy) as previously described [10] with slight modifications (see Appendix A).

### 2.2. Body Mass Index Calculation and Subgroup Analysis

The BMI is a convenient scale for evaluating human thinness and fatness, and sheep phenotyping has also begun to use BMI in recent years [11,12]. Thus, we preliminarily considered it could serve as an indicator of body growth and development in sheep research. Human BMI is defined by the formula BMI  =  weight (kg)/[height (m)]^2^. Compared with other weight/height ratios (W/H, W/H^3^, H/W^1/3^), this formula is preferred because it is relatively independent of height and correlates highly with weight and fatness [13]. For the animal models, the BMI was calculated as BMI = body weight (kg)/[body length (m)]^2^ [11], and we adopted the same definition for sheep BMI (weight (kg)/[length (m)]^2^).

**Figure A2:**
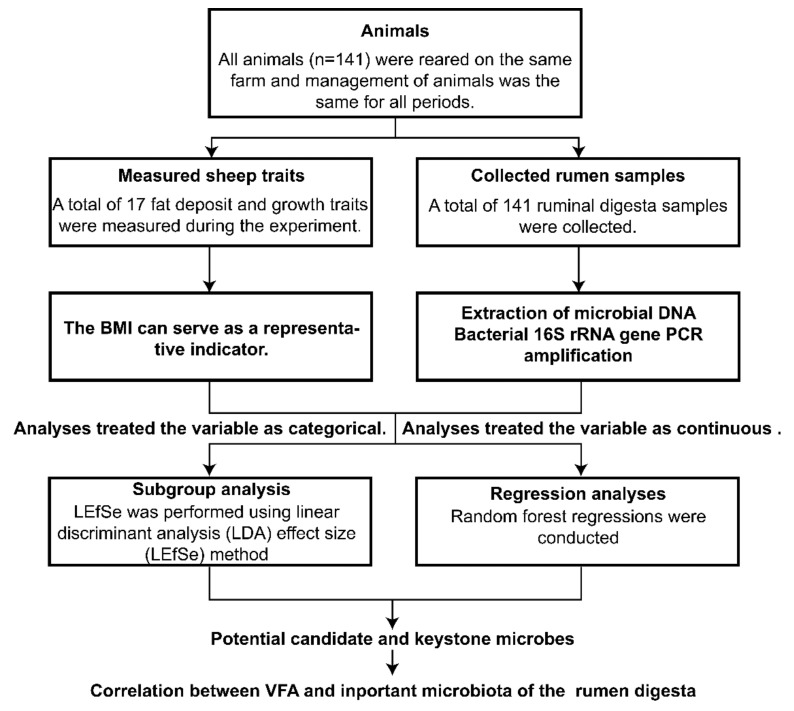
A brief flowchart of our study.

In this study, correlation analysis was used to determine the relationships among fat deposition phenotypes. Its results proved BMI could serve as an important representative indicator for the downstream analysis. Subsequently, we analyzed the relationship between fat deposition and the rumen microbiome from the perspectives of the classification method and the regression model (see Figure A2). For classification, all sheep were categorized into three groups based on their BMI level: Low fat deposition (LFD) (LFD <87.11; less than the means minus 0.5 SD, *n* = 40), medium fat deposition (MFD) (87.11 < MFD <97.35; between the means minus 0.5 SD and means plus 0.5 SD, *n* = 62), and high fat deposition (HFD) (HFD >97.35; means plus 0.5 SD, *n* = 39).

### 2.3. Extraction of Microbial DNA and Bacterial 16S rRNA Gene PCR Amplification

An EasyPure Stool Genomic DNA Kit (EE301-01; TransGen Biotech, Beijing, China) was used to isolate DNA from 20 g of frozen samples, following the manufacturer’s guidelines. The purity and concentration of the extracted DNA was measured using a Nanodrop 2000 instrument (Thermo, Dreieich, Germany). The 16S rRNA V3–V4 regions were amplified using the universal eubacterial primers (341F: 5-CCTAYGGGRBGCASCAG-3, 806R: 5-GGACTACNNGGGTATCTAAT-3). The amplicons were then purified using a TruSeq DNA PCR Free Preparation Kit (QIAGEN, Dusseldorf, Germany). The amplicons were sequenced on the IonS5TMXL platform (Novogene, Sacramento, CA, USA), which generated 600-bp single-ended reads.

### 2.4. Sequence Processing, OTU Clustering, and OTU Filtering

Following sequencing, MOTHUR [14] was used to assemble the raw sequences. Chimeric sequences were removed using the USEARCH Uparse v7.0.1001 software [15], based on the UCHIME algorithm [16]. The SILVA database (http://www.arb-silva.de/) (accessed on 16 December 2017) was used to align the resultant sequences [17]. The Ribosomal Database Project Classifier [17] was used to perform the taxonomic analysis of representative operational taxonomic unit (OTU) sequences at a similarity level of 97%. Based on the number of sequenced reads, we generated an OTU table by calculating the absolute abundance of each identified OTU for each sample. The data of OTU abundance were subsequently filtered, (Low count filter: At least 20% of its values should contain at least four counts [18]. Low variance filter: Percentage to remove 10% based on the Inter-quantile range [19]) and then transformed based on total sum scaling. This process reduced the total OTU number from 3757 to 705. The resulting 705 OTUs were used for the analysis.

### 2.5. Statistical Analysis

For data related to fat deposition phenotypes, descriptive statistics (means, SD, and coefficient of variation (CV)) and the comparison of groups were conducted using Python 3 (*Python* Software Foundation, Beaverton, OR) and GraphPad Prism 8 (GraphPad Software, La Jolla, CA, USA). The differences among the groups were analyzed using one-way ANOVA, and the correlation between different traits was measured by Spearman’s rank correlation test (very weak: 0.00–0.19, weak: 0.20–0.39, moderate: 0.40–0.59, strong: 0.60–0.79, and very strong: 0.80–1.0) and visualized using the R *psych* package [20]. Linear regression fit was performed to determine relations between BMI and VFA data sets (*lm4* in R) [21].

A subsequent Mantel test was conducted to determine the relationship between animal intake and microbial population. The Shannon and Simpson indexes were used to evaluate the alpha diversity of the rumen microbiota. To assess the estimated β-diversity, Bray–Curtis dissimilarities were computed between samples and then Principal coordinate analysis (PCoA) ordination was used for visualization. Beta diversity (community overlap) was also compared using PERMANOVA analyses between groups. Linear discriminant analysis (LDA) Effect Size (LEfSe) analysis was applied to identify potential microbial biomarkers between groups, using false discovery rate (FDR) values of 0.05 and an LDA threshold score of 3.0. This part of the workflow was calculated using the R package MicrobiomeAnalystR [22] and *vegan* [23].

To obtain the optimal discriminant performance of the rumen microbiota across different fat depositions, the Random Forest (RF) regression model was used to regress the relative abundances of bacterial taxa against the BMI. The RF model was performed using the R package randomForest [24], with the number of trees set to 500. Lists of bacteria ranked in order of feature importance were constructed. An assessment of feature importance was performed using IncNodePurity (a variable importance measure). Five repeats of 10-fold cross-validation, implemented using the *rfcv()* function, were used to identify the number of marker taxa. Then, a locally weighted regression (LOESS) modeled the relationship between biomarkers and BMI. To assess the effect of important microbiota on VFA, Spearman’s correlations were performed and calculated using *corr.test* in psych library (parameters: adjust  =  “BH”).

## 3. Results

### 3.1. Phenotypic Parameters and Correlations between Traits

**Figure 1:**
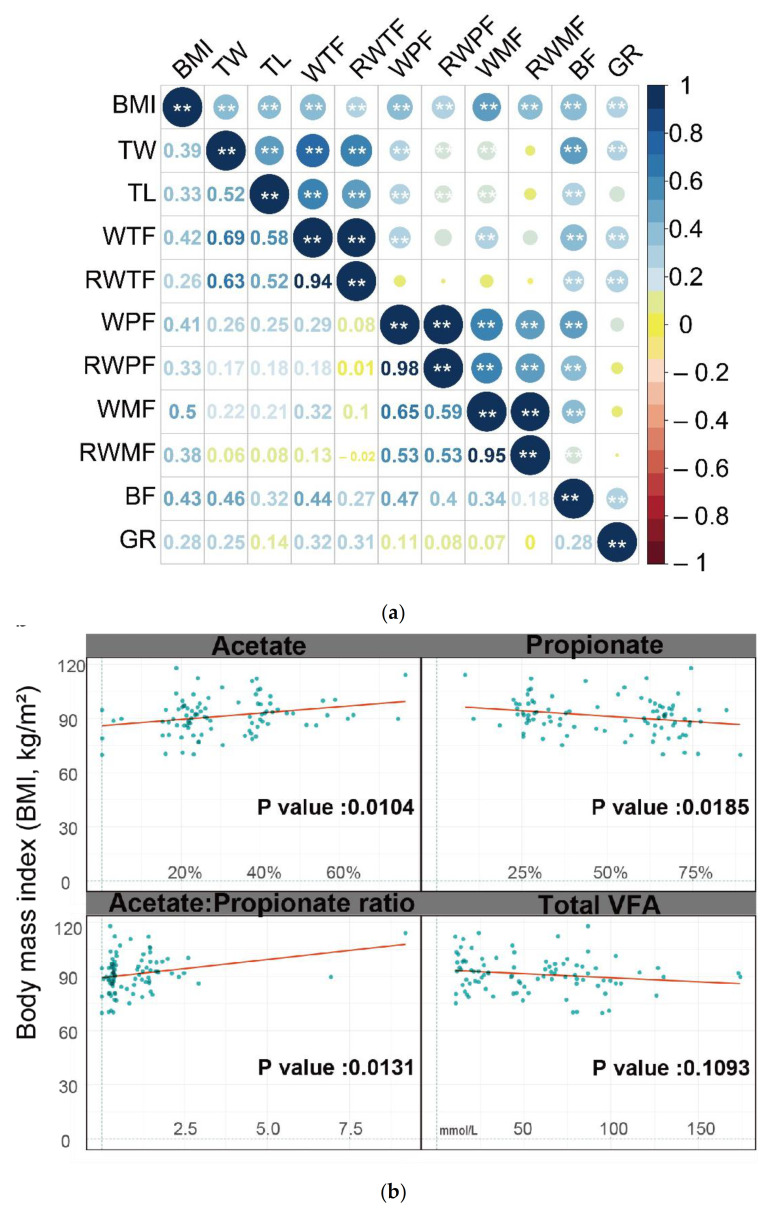

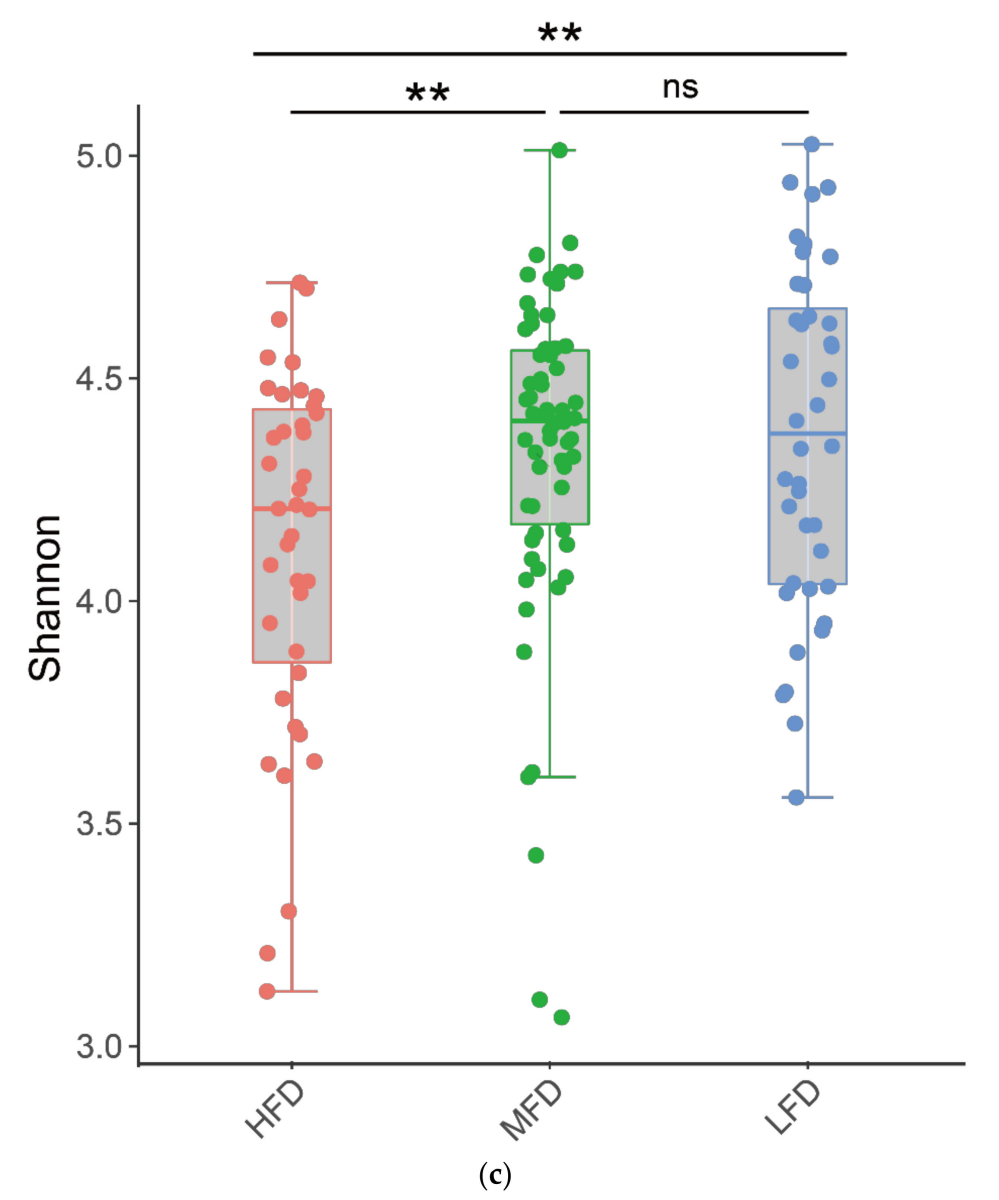

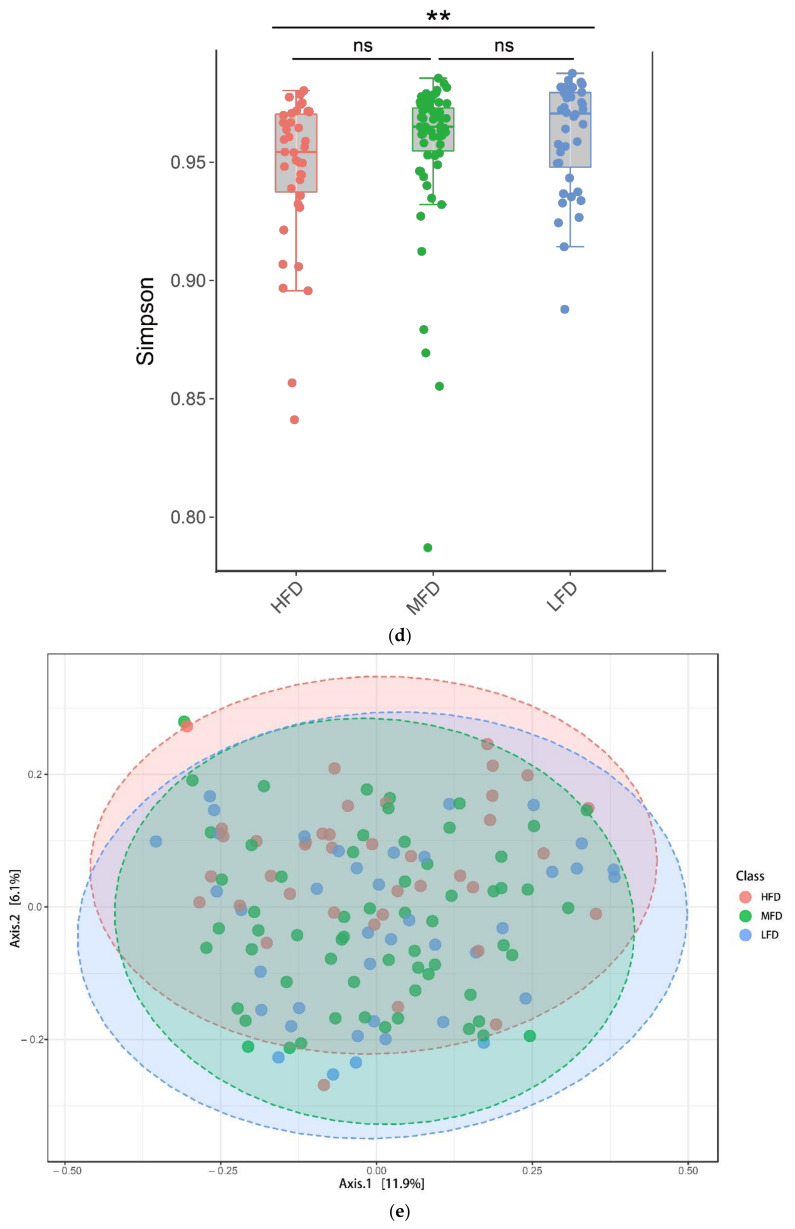
(**a**) Correlations between the analyzed phenotypic profiles of fat deposition in sheep. The size of the circle is proportional to the correlation. All traits were correlated to Body Mass Index (BMI) (0.26 < r < 0.50, 3.38 × 10^−10^ < *p* < 0.0019) and body fat thickness (0.18 < r < 0.47, 2.70 × 10^−9^ < *p* < 0.0375). ** *p* < 0.01. Body mass index (BMI); Tail width (TW); Tail length (TL); The weight of tail fat (WTF); The relative weight of tail fat (RWTF); The weight of perirenal fat (WPF); The relative weight of perirenal fat(RWPF); The weight of mesenteric fat (WMF); The realtive weight of mesenteric fat (RWMF); Thickness of backfat (BF); GR value (GR) (**b**) Linear regression fit was performed to determine relations between BMI and volatile fatty acids’ (VFAs’) data sets (for further details, see Table A2). Total VFA (mmol/L) = the sum of all individual volatile fatty acids. Single VFA (%) was expressed as relative amounts compared with total VFA concentration. Scatter plots with linear fit are shown and r and *p* values are listed; the red line shows the best-fit linear regression. To clearly compare the Shannon diversity (**c**) and Simpson diversity (**d**) (alpha-diversity) between the sheep with different levels of fat deposition, we generated boxplots to show the variation between the three groups (ns: *p* > 0.05, ** *p* < 0.01). (**e**) The PCoA plots showed no separation among these groups, which indicates BMI did not have a major effect on microbiota composition. These findings were supported by the results of the statistical analysis (Permutational Multivariate Analysis of Variance, PERMANOVA; F = 1.32; R2 = 0.02; *p* < 0.05).

Details of fat deposit traits are reported in Table 1. Figure 1a provides an overview of all correlations between the fat deposition traits, which showed a positive correlation in each comparison. The correlations between many traits were moderate to high, ranging from 0.31 (between the GR value and the relative weight of the tail fat) to 0.65 (between the weight of the mesenteric fat and the weight of the perirenal fat). All traits correlated with the BMI (0.26 < r < 0.50, 3.38 × 10^−10^ < *p* < 0.0019) and body fat thickness (0.18 < r < 0.47, 2.70 × 10^−9^ < *p* < 0.0375).

The results of ANOVA demonstrated significant differences among groups for all 11 fat deposit traits (Mean values trend: LFD < MFD < HFD) under study (Table 2). At 80 days and 180 days (the time of sacrifice), we noted a significant difference in body weight; the weight of the sheep in the HFD group was larger than that of the sheep in the LFD and MFD groups. Additionally, we noted that the animal feed intake and growth rate showed significant group differences.

The major VFAs (Acetate, Propionate, and Butyrate) accounted for nearly 90% of total VFAs in rumen. For the relationship between BMI and VFAs, data sets were fitted to the linear models (Figure 1b and Table A2). The only significant negative linear correlation was for propionate.

### 3.2. Comparing the Rumen Microbial Community Structures among Sheep with Distinct Levels of Fat Deposition

In accordance with Mantel test results, the effect of ADFI on microbial population did not reach the significance threshold (Mantel r = −0.009759, *p* = 0.6012). For this reason, the following analysis does not further consider the animal intake. We then explored the community structures of the rumen microbiotas among the FD groups using alpha and beta diversity metrics. The Simpson index was not significantly different (ANOVA test, *p* = 0.09; Figure 1d); however, the Shannon index showed differences between the groups (ANOVA test, *p* = 0.008; Figure 1c). Significant differences were found for subsequent multiple comparisons using the least significant difference (LSD) test; the Shannon and Simpson indexes were significantly higher in the LFD group than in the HFD group. The *Firmicutes/Bacteroidetes* ratio is an important parameter to evaluate the distribution of the gut flora; however, there was no significant differences in the *Firmicutes/Bacteroidetes* ratio in the present study (LFD = 1.36, MFD = 1.13, HFD = 1.03; *p* > 0.05). The results of PCoA analyses based on OTU levels were displayed as scatter plots (Figure 1e), and no significant regional differences were evident, which indicates BMI did not have a major effect on microbiota composition. The statistical analysis supported these results (PERMANOVA; F = 1.32; R^2^ = 0.02; *p* < 0.05).

### 3.3. Analysis of Differential Rumen Microbiota

**Figure 2:**
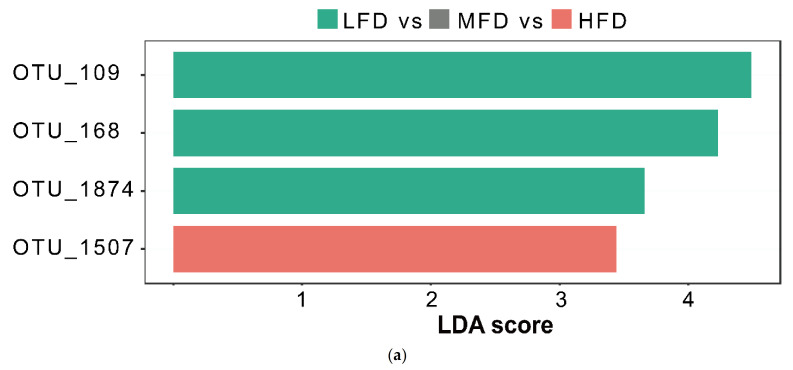

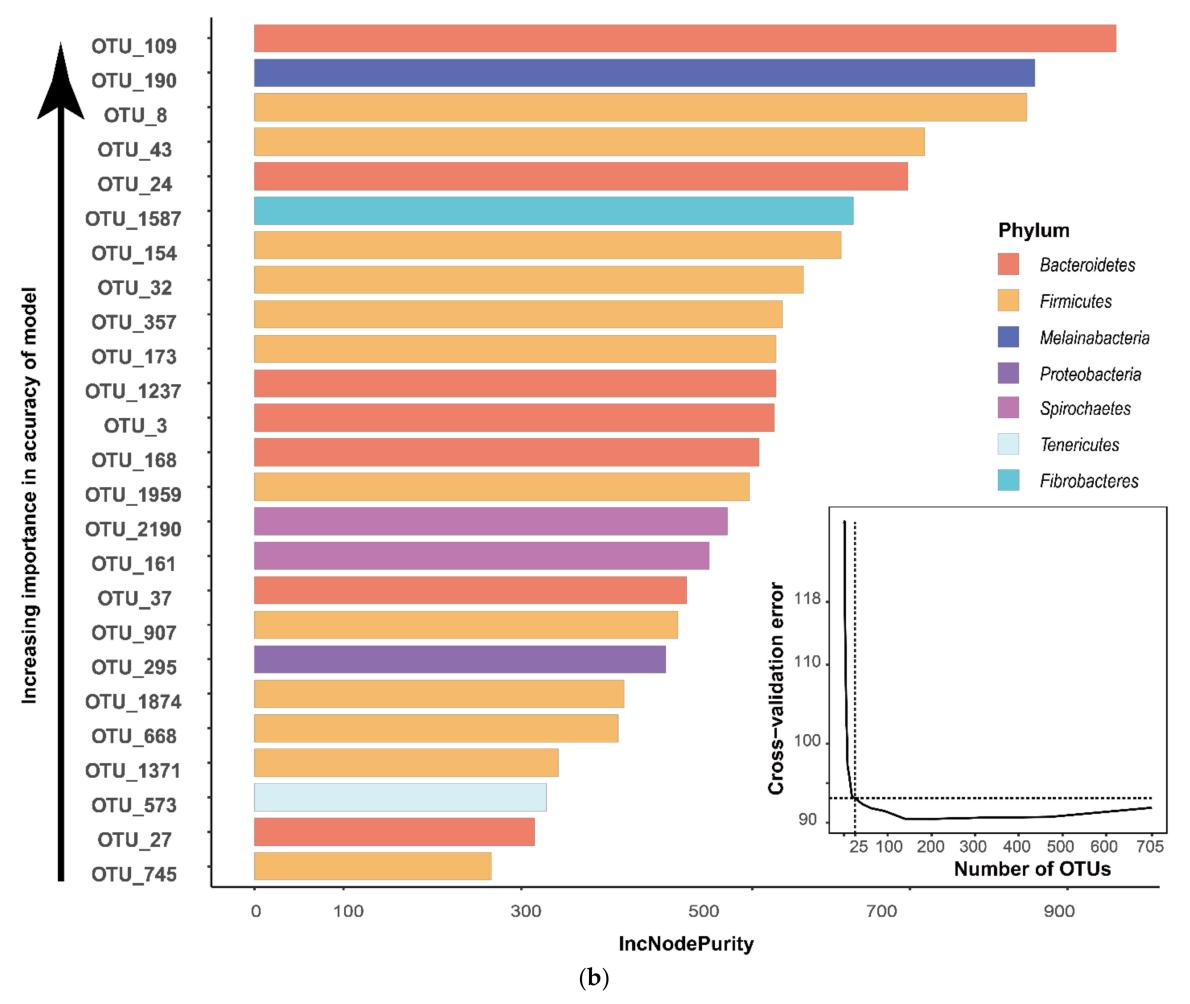

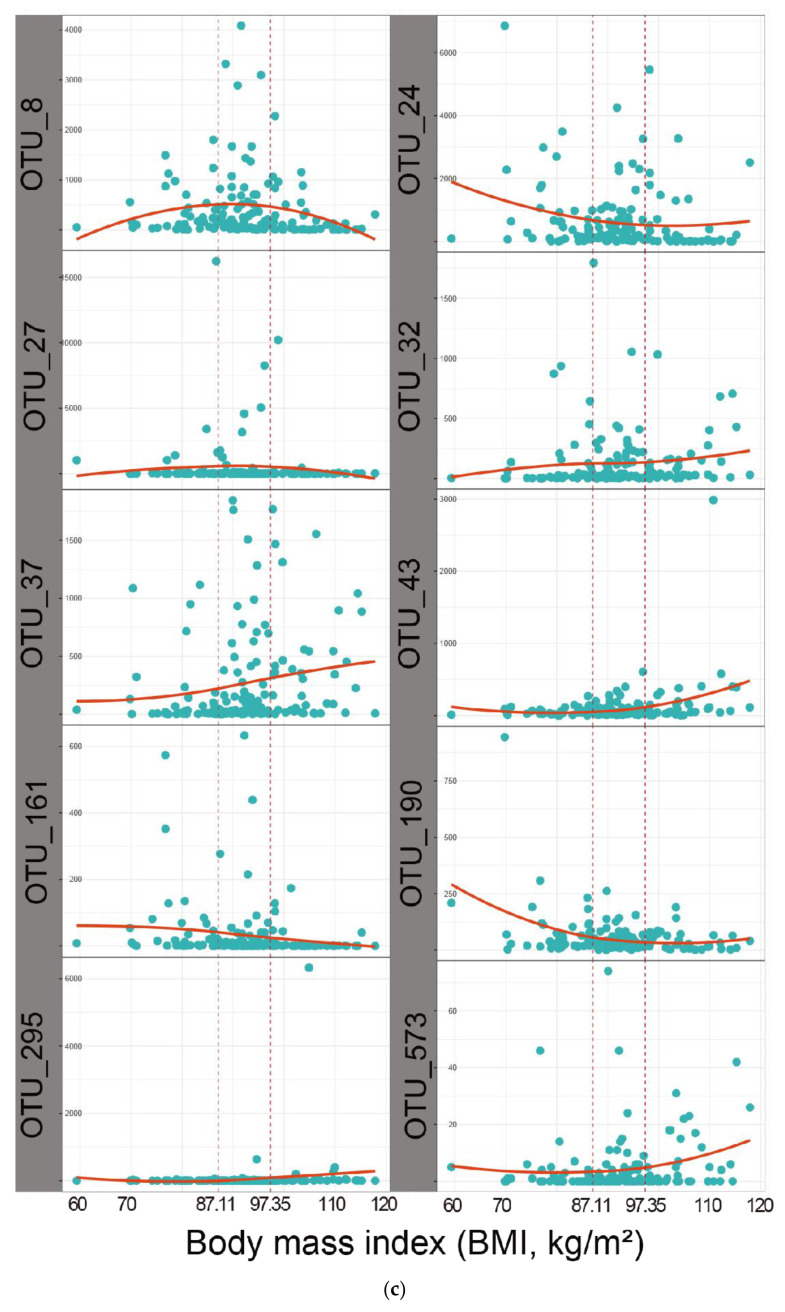
(**a**) Linear discriminant analysis (LDA) effect size (LEfSe) analyses identified rumen bacterial biomarkers of sheep with different amounts of fat deposition (LDA >3, FDR <0.05). (**b**) The top 25 biomarker bacterial classes were identified by applying Random Forest regression analysis of the relative abundance of rumen bacteria at the OTU level against BMI in the sheep. Biomarker taxa are ranked in descending order of importance according to the accuracy of the model. The insert represents 10-fold cross-validation error as a function of the number of input classes used for regression against BMI in the sheep in order of variable importance. (**c**) The locally weighted regression (LOESS) modeled the relationship between significant biomarkers and Body Mass Index (BMI). Only the microbial taxa from the non-overlap results and above the genus level were shown (10/29). The red line represents a locally weighted scatter plot (LOESS) regression curve.

LEfSe analysis was performed to identify the differentially abundant bacteria composition (Figure 2a; LDA> 3, FDR< 0.05). In the LFD group, *Prevotellaceae* of the *Bacteroidetes* (two OTUs: 109, 168) and *Lachnospiraceae* (OTU_1874) of the *Firmicutes* were identified as important microbial biomarkers. Only *unidentified*_*Ruminococcaceae* (OTU_1507) of the *Firmicutes* was enriched in the HFD group.

To avoid the bias introduced by categorization, the RF machine learning algorithm was used to regress the relative abundance of rumen bacteria against the BMI of the sheep, to establish a model that correlated the fat deposition level with the rumen microbiota composition. The model explained 15.5% of the variance in the rumen microbiota related to the fat deposition level in the sheep. Additionally, the Mantel test revealed that bacterial communities and fat deposition had a weak but significant positive correlation (*n* = 141, mantel test: r = 0.10, *p* = 0.004). To verify the relevance of bacterial communities to phenotype, some randomly selected samples (close to normal distributions) were tested, and this relationship proved, once again, significant (see Table A4). To determine which rumen bacterial taxa were the primary drivers of fat deposition in sheep, five repeats of 10-fold cross-validation were performed to evaluate the important bacteria. When we used 25 OTUs, the number of classes against the cross-validation error curve stabilized; therefore, these 25 OTUs were defined as biomarkers in the model (Figure 2b). The taxonomic profiles of the detected 25 biomarker OTUs are summarized in Table A3. Among the results, three OTUs were assigned to *Prevotellaceae* and three to *Lachnospiraceae*. At the genus level, we detected *Oribacterium* and *Succinivibrio*. In addition, eight bacteria species (in 29 biomarkers, see Table A3) were associated with sheep BMI. In these two approaches, three overlapping markers were identified. Subsequently, locally weighted regression curve fitting was applied to visualize the trends between biomarkers and BMI. In this analysis, only the microbial taxa from the non-overlap results and above the genus level were shown.

### 3.4. Correlation between VFA and Significant Biomarkers of Rumen Digesta

**Figure 3:**
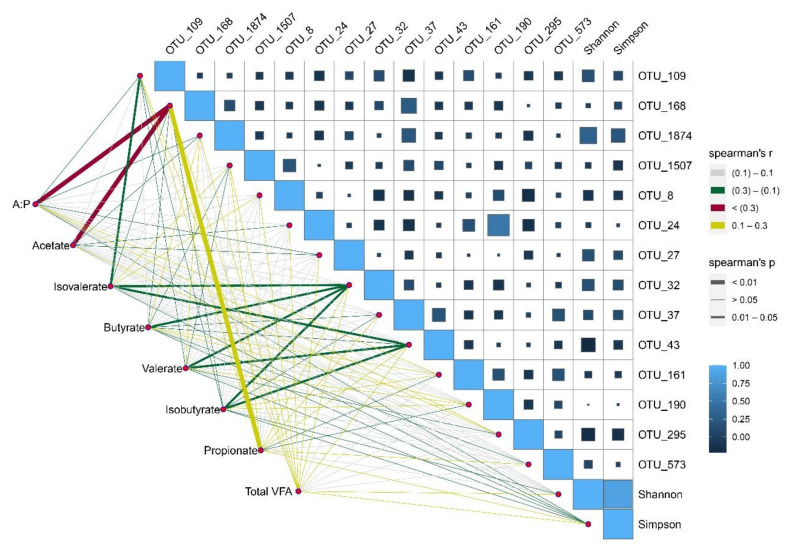
Heatmap showing the Spearman’s correlation coefficients among important ruminal microbes and eight ruminal fermentation parameters. Distance correlation plots of 14 OTUs and the ruminal fermentation parameters. Note: (value) = Negative values.

We further analyzed the correlations between the important ruminal microbes and ruminal fermentation parameters and found seven clinical indicators were closely related to the important ruminal microbes (Figure 3, the absolute values of spearman’s r > 0.1 and *p* < 0.05). The *Prevotellaceae* were positively correlated with isovalerate (r = −0.25). The correlations were observed between *Prevotellaceae* and three parameters: *Prevotellaceae* had relatively higher negative correlations with acetate: propionate (r = −0.32) than with acetate (r = −0.3) and significant positive correlation with propionate (r = 0.28). Of the identified bacterial biomarkers by the random forest regression model, both *Ruminococcaceae* and *Oribacterium* were negatively associated with isobutyrate, isovalerate, and valerate. Additionally, a negative correlation between *Ruminococcaceae* and butyrate (r = −0.26) was observed.

## 4. Discussion

As the main body reservoir for energy in animals, fat is mainly stored in the subcutaneous, intermuscular, intramuscular, and abdominal omental visceral adipose tissue. The deposition intensity of fat throughout the body varies by body part. Sheep fat deposition shows obvious biological characteristics of local deposition. Especially in fat-tailed sheep, the fat deposition intensity is greater in the tail compared with that in other body parts [25]. Our results showed that in fat-tailed lambs, the tail fat, perirenal fat, and mesenteric fat proportions were 2.87, 1.23, and 2.37%, respectively. However, no significant association was observed between the fat depositions among those body parts. This suggested that the fat deposition of a single body part cannot fully reflect the fat deposition level of the whole body. Thus, it is necessary and important to evaluate the fat deposition level of the whole body by acquiring fat deposition phenotypes of representative and credible multiple parts or to use a comprehensive index. Obesity is defined as an excess of body fat. In humans, the BMI has been the most widely used parameter to assess and classify the grade of obesity, based on height and weight. The BMI also has been used to assess of the level of fat deposition in farm animals [26]. Correlation analysis in a large study population showed that the BMI had a higher and more significant positive correlation with all the fat deposition phenotypes and can be used as a comprehensive index to evaluate fat deposition in sheep.

Based on what was mentioned above, in the present study animals were identified based on their BMIs in the subsequent analyses of regression and classification. Subgroup analyses indicated significant differences between groups for all the fat deposition traits studied. This result further illustrated the feasibility of using the BMI as an indicator to evaluate the fat deposition level, which formed the basis for subsequent analysis. Meanwhile, the results also showed that the ADFI and ADG were significantly higher in the HFD group, which implied that sheep with a faster growth rate might acquire a high fat content. In animals, a high growth rate mainly results from a high appetite, which might lead to higher fat deposition [27].

In ruminants, the rumen is host to a large and complex microbial community that has important functions in a variety of vital processes, such as immune development and carbohydrate metabolism. Importantly, the rumen microbiota produces VFAs and microbial proteins that provide more than 70% of the required energy and 60% of non-ammonia nitrogen to ruminants [28]. Thus, alterations to the composition and diversity of the rumen microbiota are believed to be important to the health and productivity of ruminants. In the present study, VFA profile determined rumen metabolic difference among different BMI animals. Our findings showed acetate and propionate mainly affect BMI differently, and acetate: propionate also is associated with fat deposition level. This suggests that VFAs are implicated in the regulation of energy storage through multiple mechanisms. Acetate plays an extremely critical role in fat synthesis, providing a parallel pathway for CoA production for lipogenesis, and resulted in its increased relative abundance in high BMI. Propionate is another main intermediate of interest as it is the precursor of gluconeogenesis [29]. Ruminal propionate output levels increase and hepatic gluconeogenic flux increases, which improve energy balance. The heightened basal metabolism and energy consumption in the animal increased the gluconeogenesis pathway, which might contribute to reducing the fat deposition level. This result of acetate: propionate also indirectly supported the above conjecture. It is important to mention that, in this study, all animals were fed with the same high-concentrate diet and it was what was driving the increase in the proportion of propionate and the decrease in the ratio of acetate to propionate in rumen. In addition, ruminant may be affected by short-term fasting to result in reduced levels of total VFA; however, short-term fasting was only slightly influenced by the microbiota structure [30].

In the present study of fat-tail sheep, to identify the specific members of the rumen microbiota linked to BMI, we analyzed datasets from the perspective of a classification and regression (RF regression model), respectively. For classification, the data was analyzed using subgroup analyses, and the results showed that a decrease in the richness and diversity of the rumen microbiota and changes to its overall composition were associated with fat deposition. Moreover, in conjunction with the Mantel test and the variance explained in the regression model, we further hypothesized that, in sheep, the rumen microbial composition would affect energy harvest from the diet and energy storage in the host.

Eventually, Of the 29 OTUs identified by both methods (LEfSe analysis and RF Regression), three shared OTUs were identified that corresponded to the *Prevotellaceae* and *Lachnospiraceae* families. Increases in important VFA-producing bacteria in the high-fat animals might also promote host energy harvest and, ultimately, the accumulation of fat in adipose tissue. Interestingly, the current study also found that *Prevotellaceae* were positively correlated with propionic acid and correlated negatively with acetate, indicating their potential role as bacterial biomarkers for fat deposition. In *Prevotellaceae*, we also identified a potentially novel species, *rumen_bacterium_R-9*, which makes us believe that some important bacterial genera under this family are associated with animal fat deposition phenotype. The *Lachnospiraceae* family was the main butyrate producers in the rumen and intestines of ruminants. Butyrate can increase AMPK activity and further increase energy consumption and degradation of lipids [31]. *Christensenellaceae* [32] and *Rikenellaceae* [33] also belong to the butyrate-producing bacteria and are the families consistently associated with adiposity. This was the first demonstration of the effect of *Christensenellaceae* on BMI of the ruminants. Species belonging to the *Christensenellaceae* family played a vital role in maintaining the structure and function of the rumen. Previously, it was also reported to be related to changes in rumen pH [34], suggesting *Christensenellaceae* might regulate energy collection by regulating rumen fermentation.

Although both isobutyrate, isovalerate, and valerate are negatively correlated with *Ruminococcaceae* and *Oribacterium*, judged from the fit of importance variables, the BMI levels showed an upward trend with increasing *Ruminococcaceae* and *Oribacterium*. To the best of our knowledge, *Ruminococcaceae* were involved in the digestion of fiber [35], and the role of *Oribacterium* has not been reported in the rumen. Thus, the exact role of VFAs on the animal adipogenesis and their correlation with the microbial community still need in-depth study. It is interesting to notice that *Melainabacteria*, which was identified and named by metagenomic approach methods in 2013, could produce B and K vitamins, making it act on the host itself [36]. While the specific details of those vitamins in sheep fat deposition are not known, this may give some hints that lean animals may require more essential nutrients to be involved in the basic physiological processes throughout the body. We also observed that *Mollicutes* increased with increasing BMI. Although the functions of these bacteria are unknown, many members of these families are important for animal health because they colonize mucosal surfaces and cause long-lasting and common, but mostly self-limiting, infections [37]. As one of the major species of the *Selenomonas* genus, *Selenomonas ruminantium* has been reported to be an important propionic acid-forming bacterium that participates in the rumen succinic acid pathway [38]. In our results, another reported bacterial species was *Treponema_bryantii* (*T. bryantii*). *T. bryantii* was originally isolated from bovine rumen fluid and may be involved in the degradation of soluble fibers [39].

There are certain limitations worth mentioning. The relatively small sample size of rumen functional measures was limited and only 63.83% of the total sample size participated in the VFA profile, thus reducing the study relevance. To verify and to clarify the exact roles of these microbial populations, further deep research (such as meta-omics approaches) is required.

## 5. Conclusions

In conclusion, we demonstrated that BMI was associated with a range of fat deposition traits and it can be used as an important predictor of fat parameters in sheep. Furthermore, the rumen microbiota was involved in sheep fat deposition to some extent. Rumen microbiota in low fat deposition animals have higher richness and evenness than high fat deposition animals. Moreover, we also investigated the effect of rumen microbiota on fat deposition from the perspectives of the classification method and the regression model. We identified one phylum, one class, one order, eight families, three genera, and eight species that were potentially associated with fat deposition in rumen microbiota. Our results preliminary suggest that it is possible to develop nutritional strategies to control fat deposition through manipulating rumen microbiota.

## Figures and Tables

**Table 1:** The descriptive statistics’ results of fat deposit traits in sheep.

Fat Deposit Traits	*n*	Mean	SD	CV ^3^ (%)
BMI ^1^ 180 d, kg/(m^2^)	141	92.23	10.25	11.11
Tail width 180 d, cm	141	18.50	2.23	12.08
Tail length 180 d, cm	141	19.31	2.83	14.67
The weight of tail fat, kg	141	1.30	0.43	33.22
The relative weight of tail fat, %	141	2.87	0.77	26.82
The weight of perirenal fat, kg	141	0.57	0.28	49.70
The relative weight of perirenal fat, %	141	1.23	0.52	42.70
The weight of mesenteric fat, kg	141	1.08	0.36	33.71
The relative weight of mesenteric fat, %	141	2.37	0.65	27.52
Thickness of backfat, mm	141	23.99	5.77	24.04
Rib thickness (GR value) ^2^, mm	141	15.61	4.35	27.85

^1^ BMI = Body Mass Index. ^2^ The rib thickness represents the fat content of the carcass, based on the soft tissue depth at the GR site, which was present over the 12th rib, at 110 mm away from the midline (see Figure A1). ^3^ CV = coefficient of variation (SD/mean).

**Table 2:** Differences in sheep fat deposition phenotypes and growth performance between the groups.

Trait	Groups			SE	*p* Value
	LFD	MFD	HFD		
No. of animals	40	62	39		
Fat Deposit Traits					
BMI ^1^ 180 d (BMI), kg/m^2^	80.42 ^C^	92.04 ^B^	104.64 ^A^	0.863	<0.001
Tail width 180 d (TW), cm	17.78 ^b^	18.31 ^b^	19.54 ^a^	0.188	0.001
Tail length 180 d (TL), cm	18.00 ^b^	19.60 ^a^	20.21 ^a^	0.239	0.001
The weight of tail fat (WTF), kg	1.09 ^c^	1.31 ^b^	1.51 ^a^	0.036	<0.001
The relative weight of tail fat (RWTF), %	2.63 ^b^	2.89 ^a,b^	3.08 ^a^	0.065	0.028
The weight of perirenal fat (WPF), kg	0.41 ^b^	0.58 ^a^	0.69 ^a^	0.024	<0.001
The relative weight of perirenal fat (RWPF), %	0.98 ^b^	1.28 ^a^	1.41 ^a^	0.044	<0.001
The weight of mesenteric fat (WMF), kg	0.83 ^c^	1.08 ^b^	1.33 ^a^	0.031	<0.001
The relative weight of mesenteric fat (RWMF), %	1.98 ^c^	2.41 ^b^	2.72 ^a^	0.055	<0.001
Thickness of backfat (BF), mm	21.55 ^b^	23.73 ^b^	26.90 ^a^	0.486	<0.001
Rib thickness (GR value) ^2^, mm	14.15 ^b^	15.94 ^a^	16.59 ^a^	0.366	0.032
Growth Performance					
BW 80 d, kg	16.75 ^c^	18.95 ^b^	21.99 ^a^	0.32	<0.001
BW 180 d, kg	41.44 ^c^	45.74 ^b^	50.69 ^a^	0.507	<0.001
Live weight before slaughter, kg	40.88 ^c^	44.86 ^b^	49.01 ^a^	0.472	<0.001
Body Length 180 d, cm	71.65 ^b^	70.40 ^a,b^	69.62 ^a^	0.307	0.042
ADFI ^3^ 80 d–180 d, kg/d	1.43 ^c^	1.53 ^b^	1.67 ^a^	0.017	<0.001
ADG ^4^ 80 d–180 d, kg/d	0.25 ^c^	0.27 ^b^	0.29 ^a^	0.003	<0.001

^1^ BMI = Body Mass Index. ^2^ The rib thickness represents the fat content of the carcass, based on the soft tissue depth at the GR site, which was present over the 12th rib, at 110 mm away from the midline (see Figure A1). ^3^ ADFI, average daily feed intake. ^4^ ADG, average daily gain. (Least significant difference *t*-test; a, b, c: *p* < 0.05; A, B, C: *p* < 0.01).

## Data Availability

The sequence files determined in the present study were deposited at the Sequence Read Archive (SRA; http://www.ncbi.nlm.nih.gov/Traces/sra/ (accessed on 16 December 2017); SRA accession number: PRJNA611791; Release date 7 December 2022).

## Appendix A

### Appendix A.1. Supplementary Methods: Volatile Fatty Acid Analysis

The concentration of volatile fatty acids (VFAs) in rumen chyme was measured using a gas chromatographic method as previously described with slight modifications (Erwin et al., 1961). For volatile fatty acids’ determination, ruminal fluid was centrifuged for 10 min at 5400 rpm (centrifugal radius: 14.5 cm, relative centrifugal force: 4731 g), and 1.0 mL was mixed with 0.2 mL of a 25% (*w*/*v*) metaphosphoric acid mixture (2-ethylbutyric acid as the internal standard, 2 g/L). The mixture was incubated for 30 min at 4 °C and centrifuged (10,000 rpm, centrifugal radius: 5 cm, relative centrifugal force: 5595 g) for 10 min at 4 °C. The supernatant was carefully collected and filtered through a 0.45-μm filter (0.45-μm Syringe Filters). The clear supernatant was transferred to a vial for gas chromatography. Volatile fatty acid values were determined using a TRACE-1300 series GC ultra-gas chromatograph (Thermo Scientific, Milan, Italy). The specific parameters were as follows.

The capillary chromatographic column was DB-FFAP (15 m × 0.32 mm × 0.25 µm). The sample injection volume was 1 μL. Split mode was applied with 50:1 split ratio. The temperatures of both the injection port and the detector were kept constant at 240 °C. The flow rates were: hydrogen flame gas, 35 mL/min; nitrogen carrier gas, 20 mL/min; and airflow, 350 mL/min. Temperature was held at 50 °C (5 min), raised to 190 °C (2 min) at a rate of 25 °C/min, increased to 200 °C (5 min) at a rate of 10 °C/min, and then again to 220 °C (5 min) at a rate of 10 °C/min.

**Table A1:** Diet information for animal experiments (air-dry basis).

Ingredient Composition (% as fed)		Chemical Composition	
Corn	32.5	Dry matter (DM) [%]	88.78
Corn germ meal	18	Crude protein (CP) [%]	13.09
Corn stalks	12	Digestible energy [MJ/kg]	11.11
Corn hulls	11.2	Neutral detergent fiber (NDF) [%]	27.08
Corn cob	8	Acid detergent fiber (ADF) [%]	13.99
Soybean meal	5	Crude fiber (CF) [%]	9.78
Cotton meal	5		
Molasses	3.3		
Bentonite	1.5		
Baking soda	1		
Stone powder	0.8		
Expanded Urea	0.5		
Premix	0.5		

Notes: Dry matter (DM; Method 934.01), crude protein (CP; Method 954.01), and crude fiber (CF; Method 962.09) in the feeds were assayed as described by the Association of Official Analytical Chemists (AOAC, 1990). Neutral detergent fiber (NDF) and acid detergent fiber (ADF) were analyzed according to the procedures of Van Soest et al. (Van Soest et al., 1991). Digestible energy was calculated from data provided by the Feed Database of China (the tables of feed composition and nutritive values in China (2016, 15th edition)).

**Table A2:** The linear models for the association between Body mass index (BMI) and volatile fatty acids (VFAs).

	Regression Coefficients(β)	Std. Error	*p* Value
Acetate	17.57	6.71	0.0104
Propionate	−12.05	5.02	0.0185
Isobutyrate	134.57	78.1	0.0884
Butyrate	7.74	11.88	0.5164
Isovalerate	13.74	27.67	0.6208
Valerate	−64.9	96.26	0.502
Total VFA	−0.04	0.03	0.1093
Acetate:Propionate ratio	2.02	0.8	0.0131

Total VFA = the sum of all individual volatile fatty acids (VFA).

**Table A3:** The taxonomic profiles of the detected 25 biomarker OTUs by Random Forest.

OTUs	Phylum	Class	Order	Family	Genus	Species
OTU_1237	*Bacteroidetes*	*Bacteroidia*	*Bacteroidales*	*Prevotellaceae*	*unidentified_Prevotellaceae*	*rumen_bacterium_R-9*
OTU_27	*Bacteroidetes*	*Bacteroidia*	*Bacteroidales*	*Muribaculaceae*		
OTU_3	*Bacteroidetes*	*Bacteroidia*	*Bacteroidales*	*Prevotellaceae*		
OTU_109	*Bacteroidetes*	*Bacteroidia*	*Bacteroidales*	*Prevotellaceae*		
OTU_168	*Bacteroidetes*	*Bacteroidia*	*Bacteroidales*	*Prevotellaceae*		
OTU_24	*Bacteroidetes*	*Bacteroidia*	*Bacteroidales*	*Rikenellaceae*		
OTU_37	*Bacteroidetes*	*Bacteroidia*	*Bacteroidales*			
OTU_1587	*Fibrobacteres*	*Fibrobacteria*	*Fibrobacterales*	*Fibrobacteraceae*	*Fibrobacter*	*bacterium_NC3008*
OTU_43	*Firmicutes*	*Clostridia*	*Clostridiales*	*Lachnospiraceae*	*Oribacterium*	
OTU_173	*Firmicutes*	*Clostridia*	*Clostridiales*	*Lachnospiraceae*	*unidentified_Lachnospiraceae*	*bacterium_AD3010*
OTU_907	*Firmicutes*	*Clostridia*	*Clostridiales*	*Lachnospiraceae*	*unidentified_Lachnospiraceae*	*Lachnospiraceae_bacterium_NK4A179*
OTU_1959	*Firmicutes*	*Clostridia*	*Clostridiales*	*Lachnospiraceae*	*unidentified_Lachnospiraceae*	*bacterium_enrichment_culture_clone_R1-3*
OTU_154	*Firmicutes*	*Negativicutes*	*Selenomonadales*	*Veillonellaceae*	*unidentified_Veillonellaceae*	*Selenomonas_ruminantium*
OTU_668	*Firmicutes*	*Negativicutes*	*Selenomonadales*	*Veillonellaceae*	*unidentified_Veillonellaceae*	*rumen_bacterium_RC-11*
OTU_8	*Firmicutes*	*Clostridia*	*Clostridiales*	*Christensenellaceae*		
OTU_357	*Firmicutes*	*Clostridia*	*Clostridiales*	*Lachnospiraceae*		
OTU_1371	*Firmicutes*	*Clostridia*	*Clostridiales*	*Lachnospiraceae*		
OTU_1874	*Firmicutes*	*Clostridia*	*Clostridiales*	*Lachnospiraceae*		
OTU_32	*Firmicutes*	*Clostridia*	*Clostridiales*	*Ruminococcaceae*		
OTU_745	*Firmicutes*	*Negativicutes*	*Selenomonadales*	*Veillonellaceae*	*g__unidentified_Veillonellaceae*	
OTU_190	*Melainabacteria*					
OTU_295	*Proteobacteria*	*Gammaproteobacteria*	*Aeromonadales*	*Succinivibrionaceae*	*Succinivibrio*	
OTU_2190	*Spirochaetes*	*Spirochaetia*	*Spirochaetales*	*Spirochaetaceae*	*unidentified_Spirochaetaceae*	*Treponema_bryantii*
OTU_161	*Spirochaetes*	*Spirochaetia*	*Spirochaetales*	*Spirochaetaceae*		
OTU_573	*Tenericutes*	*Mollicutes*				

**Table A4:** The results of Mantel test validation.

Sample Size (Randomly Selected)	Number of Replications	Correlation Coefficient (r)				*p* Value
		Mean	Max	Min	Sd	
20.00	120.00	0.23	0.38	0.17	0.05	<0.001
30.00	110.00	0.20	0.30	0.15	0.04	<0.001
50.00	90.00	0.15	0.24	0.10	0.03	<0.001
70.00	70.00	0.13	0.22	0.09	0.03	<0.001
80.00	60.00	0.12	0.19	0.08	0.03	<0.001
90.00	50.00	0.12	0.16	0.08	0.02	<0.001
100.00	40.00	0.11	0.15	0.07	0.02	<0.001
110.00	30.00	0.10	0.15	0.07	0.02	<0.001
120.00	20.00	0.10	0.16	0.07	0.02	<0.001
130.00	10.00	0.10	0.12	0.09	0.01	<0.001
140.00	1.00	0.10	--	--	--	<0.001

### Appendix A.2. Appendix A References

1. Erwin, E.S.; Marco, G.J.; Emery, E.M. Volatile fatty acid analyses of blood and rumen fluid by gas chromatography. *J. Dairy Sci.* 1961, *44*, 1768–1771.
2. AOAC. *Official Methods of Analysis*, 15th ed.; Association of Official Analytical Chemists: Arlington, VA, USA, 1990.
3. Van, Soest, P.J.; Robertson, J.B.; Lewis, B.A. Methods for dietary fiber, neutral detergent fiber, and nonstarch polysaccharides in relation to animal nutrition. *J. Dairy Sci.* 1991, *74*, 3583–3597.
